# Towards preventing exfoliation glaucoma by targeting and removing fibrillar aggregates associated with exfoliation syndrome

**DOI:** 10.1186/s12951-022-01665-6

**Published:** 2022-10-27

**Authors:** Mehdi Ghaffari Sharaf, Kosala D. Waduthanthri, Andrew Crichton, Karim F. Damji, Larry D. Unsworth

**Affiliations:** 1 Department of Chemical and Materials Engineering, University of Alberta, Edmonton, AB Canada; 2 Department of Ophthalmology, University of Calgary, Calgary, Canada; 3 Department of Ophthalmology and Visual Sciences, University of Alberta, Edmonton, AB Canada

**Keywords:** Exfoliation syndrome, Phage display, Peptide, Targeting, Magnetic particle

## Abstract

Exfoliation syndrome presents as an accumulation of insoluble fibrillar aggregates that commonly correlates with age and causes ocular complications, most notably open-angle glaucoma. Despite advances in understanding the pathogenesis and risk factors associated with exfoliation syndrome, there has been no significant progress in curative pharmacotherapy of this disease. It is thought that the ability to target the fibrillar aggregates associated with exfoliation may offer a new therapeutic approach, facilitating their direct removal from affected tissues. Phage display techniques yielded two peptides (LPSYNLHPHVPP, IPLLNPGSMQLS) that could differentiate between exfoliative and non-affected regions of the human lens capsule. These peptides were conjugated to magnetic particles using click chemistry to investigate their ability in targeting and removing exfoliation materials from the anterior human lens capsule. The behavior of the fibrillar materials upon binding to these magnetic particles was assessed using magnetic pins and rotating magnetic fields of various strengths. Ex vivo studies showed that the magnetic particle-peptide conjugates could generate enough mechanical force to remove large aggregates of exfoliation materials from the lens capsule when exposed to a low-frequency rotating magnetic field (5000 G, 20 Hz). Biocompatibility of targeting peptides with and without conjugated magnetic particles was confirmed using MTT cell toxicity assay, live/dead cell viability assay, and DNA fragmentation studies on primary cultured human trabecular meshwork cells. This is a novel, minimally invasive, therapeutic approach for the treatment of exfoliation glaucoma via the targeting and removal of exfoliation materials that could be applied to all tissues within the anterior segment of the eye.

**Supplementary Information:**

The online version contains supplementary material available at 10.1186/s12951-022-01665-6.

## Introduction

Exfoliation syndrome (XFS, a.k.a. pseudoexfoliation) is known as the most common identifiable cause of glaucoma, affecting ~ 70 million individuals in the world [1]. XFS is commonly considered an age-related disease that significantly affects the homeostasis of the human eye through the formation of small deposits of white materials throughout the anterior. Although their precise composition is unknown, these deposits are considered to be amyloid-like fibrils, with varied thicknesses, embedded in a fibrillogranular matrix of glycoprotein-proteoglycan crosslinks [2–6]. When found in the trabecular meshwork, these fibrillar deposits are thought to impede the outflow of aqueous humor and cause large fluctuations in intraocular pressure (IOP) that ultimately leads to irreversible blindness, namely, exfoliation glaucoma (XFG) [7–9]. Despite best clinical practice, IOP levels for patients with XFG are unpredictable and hard to control [8]. XFS has been also been causally related to lens subluxation, zonular instability, blood-aqueous barrier impairment, and several intraoperative and postoperative complications that occur during ocular treatments [10, 11]. Moreover, there is a body of evidence suggesting that XFS is a systemic disease, which presents in blood vessels, lungs, skin, gallbladder, heart, meninges, and it is a potential risk factor for other clinical complications such as coronary artery disease, cerebrovascular disease, and renal artery stenosis [12–14].

XFS materials are not removed through normal regulatory processes necessary for ocular homeostasis and, despite some success in elucidating pathomechanisms, curative pharmacotherapy to prevent, break down, or remove these materials has not yet been achieved. Treating this disease necessitates the ability to preferentially target the fibrillar structures related to XFS within the in vivo context. Compared to the other targeting approaches, peptide-based therapeutic strategies have benefited from a lower immunogenicity profile, higher binding affinity, and increased specificity related to the small peptide molecules relative to other drug compounds [15, 16]. Phage display is a powerful technique for screening a library of random amino acid sequences to identify peptides that specifically, and robustly, bind to substrates with an antibody-like affinity; a strategy used to identify peptides that bind Alzheimer's disease plaques [17, 18]. Moreover, phage display provides high-throughput screening of random peptide libraries without a priori knowledge of the target properties, which is pertinent as the physicochemical properties of these fibrillar structures are ill-defined.

Many types of nanomaterials have been applied to biomedical problems [19, 20]. Magnetic particles (MPs) have been employed for diagnosis and treatment applications as they have shown relatively good biocompatibility and can be directed to specific sites in the body using external magnetic fields. Furthermore, when exposed to an external low-frequency rotating magnetic field, similar MPs have generated mechanical forces that have been utilized for a variety of biomedical applications [21]. Iron oxide particles are relatively easy to functionalize and compared to pure metals, they are less sensitive to oxidation [22, 23]. Besides, compared to other magnetic materials including nickel and cobalt, iron oxide-based magnetic particles have higher biocompatibility which makes them more favorable candidates for biomedical applications [24].

Herein, we have used ex vivo phage display to identify high-affinity peptides that are specific to XFS fibrils. All phage binding experiments were conducted using extracted human aqueous humor (hAH) so as to mimic the physiological pH and solution properties that are crucial to molecular interactions (i.e., ion, protein, osmolarity) [25]. Identified XFS-targeting peptides were conjugated to spherical magnetic particles (1 µm dia.) and evaluated for their ability to bind XFS materials and liberate them under an induced magnetic field. Conjugation of alkyne-modified peptides to azide-functionalized MPs was confirmed using surface zeta potential measurements, Fourier-transform infrared spectroscopy (FTIR), and competitive labeling of MPs. The targeting capability of peptide modified MPs was evaluated against their scrambled sequences for binding to human lens capsules with and without XFS materials. Cellular uptake of MP-peptide conjugates was studied using electron microscopy. Cytotoxicity of MP-peptide complexes was evaluated using live/dead cell viability assay, MTT assay, and DNA fragmentation. The effect of a magnetized pin or a rotating magnetic field on the removal of XFS materials bound to peptide modified MPs was evaluated using XFS lens capsules (ex vivo). It was found that peptide-modified MPs were able to remove XFS materials from a wide range of patient samples when an external magnetic field was applied. It is thought that these engineered materials will provide a minimally invasive therapeutic strategy for treating XFS that can dramatically affect the onset and/or the course of glaucoma.

## Results and discussion

### Ex vivo phage display against XFS materials

Peptide sequences that bind to XFS materials were deduced from the DNA sequences of selected phage clones. Three rounds of biopanning against XFS materials sourced from different patients yielded an enrichment of two peptides: LPSYNLHPHVPP (p-LPS) and IPLLNPGSMQLS (p-IPL) (Additional file 1: Table S1). The high degree of selectivity of these phages towards XFS materials was aided by: i. negative screening through removal of phage that bound normal lenses through negative biopanning, and ii. removal of XFS materials from the lens capsules, resulting in specific amplification of phage that only bound to these fibrils. Moreover, the robust nature of the binding was evident as samples came from a host of different patients yet the responses were similar. Finally, the use of extracted human aqueous humor at 37 °C [26] was vital to these experiments so as to maintain a solution environment (i.e., pH, ionic strength, proteins, osmolarity) that was as close as possible to the physiological environment where the targeting of fibrils would occur.

### Evaluating the targeting ability of phage-displayed peptides

**Fig. 1:**
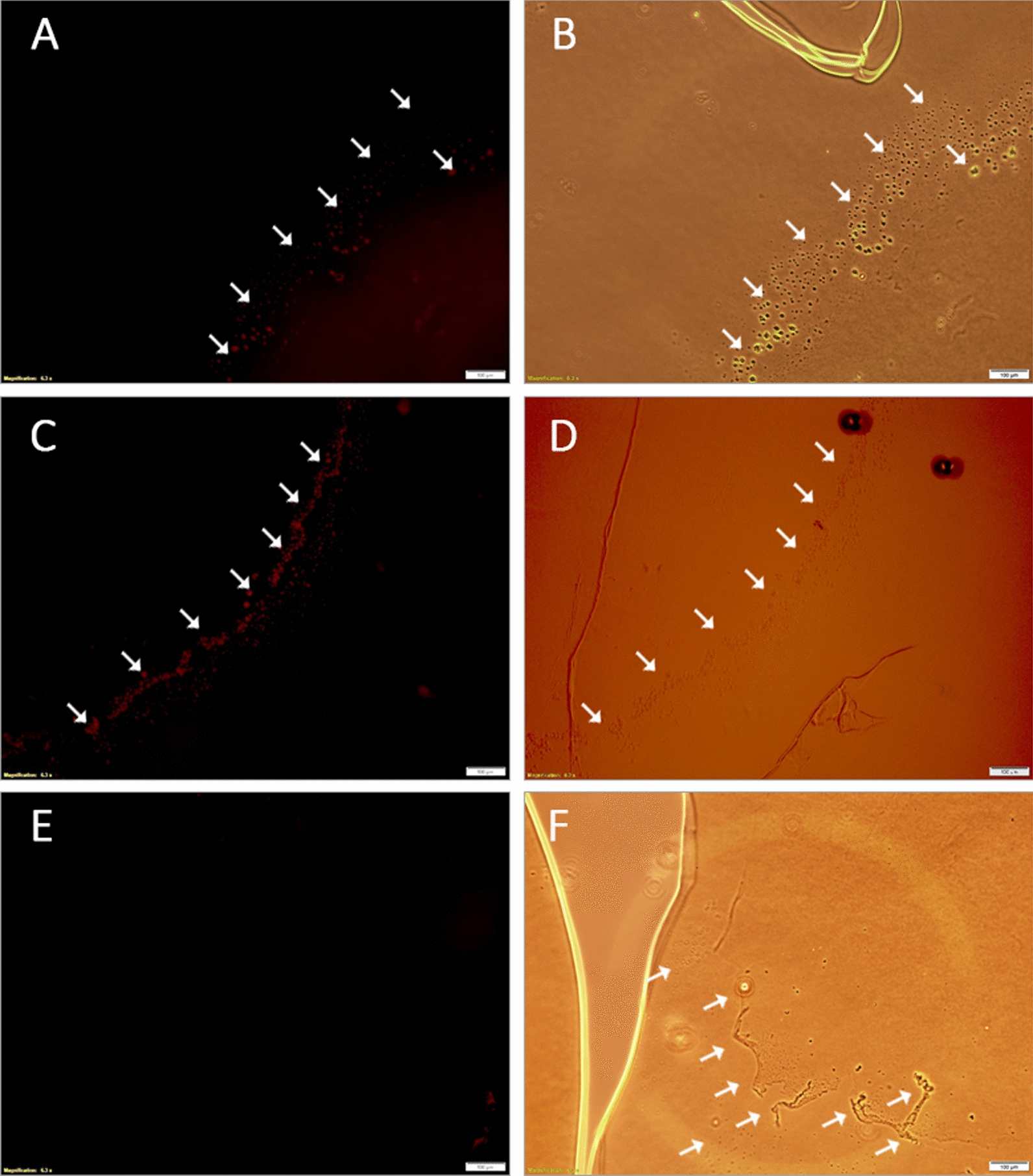
Localization of labeled phage-displayed peptides on the human lens capsule having XFS materials. M13 phages with XFS material-targeting peptides displayed on coat protein pIII and wild-type M13 phages were labeled with Cy5 fluorescent dye and incubated with human lens capsule containing XFS materials. Phages with displayed p-LPS (**A**) and p-IPL (**C**) peptides on their surface and labeled with Cy5 dye were selectively bound to the XFS materials on the lens capsule. Wild-type phages labeled with Cy5 dye (control) did not show any specific interaction with XFS materials (**E**). The presence of XFS materials on the lens capsule was confirmed in the bright field mode of the microscope (**B**, **D**, and **F**). Arrows show the exfoliative zones on the human lens capsule. (Scale bars = 100 µm)

Human lens capsules with XFS materials were stained with fluorescently labeled wild-type phages as well as phages displaying the enriched peptides. It is important to note that the conjugated fluorophore (Cy5 NHS ester dye) molecule did not interfere with phage binding to the XFS materials as it reacts with the primary amino group of lysine, which was not present in the enriched peptides. Both phage-displayed peptides (p-LPS and p-IPL) showed specific binding to XFS materials (Fig. 1A, C), where the presence of XFS materials was already confirmed using bright-field microscopy (Fig. 1B, D, F). Wild-type phages showed no noticeable interaction with XFS materials on the surface of the lens capsule (Fig. 1E). XFS materials do not cover the whole surface of the lens cuspule, meaning that labeled phages had the chance to interact with the non-XFS altered regions of the lens capsule. Phage were observed to bind only to the XFS regions of the lens capsule, further confirming their specificity towards the XFS materials.

### Peptide-particle conjugation

**Fig. 2:**
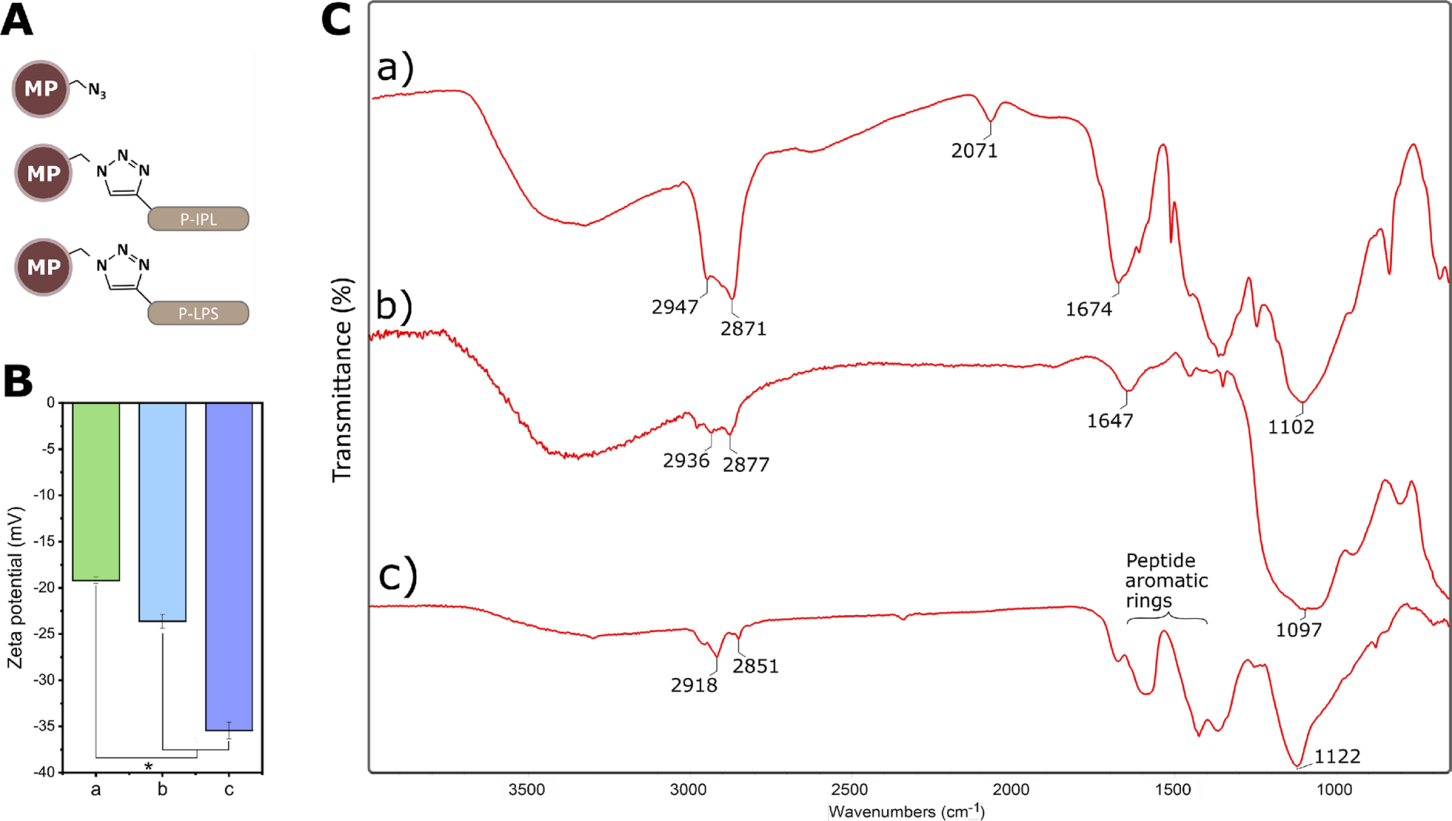
Conjugation of peptides to MPs. (**A**) Schematic illustration of MPs with and without peptide conjugates. (**B**) Surface zeta potential measurements and (**C**) FTIR spectra of MPs before and after peptide conjugation to MPs. a) Azide-functionalized MPs without peptide conjugates, b) MP-p-IPL, c) MP-p-LPS. (* represents p < 0.05, data represent mean ± 1 SD, n ≥ 3)

Conjugation of alkyne-modified peptides to azide-functionalized MPs (Fig. 2A) was confirmed with surface zeta potential measurements, Fourier-transform infrared spectroscopy (FTIR), and a competitive inhibition assay. Peptide tethering through the N-terminal domain was expected to yield an increase in negative charge on the surface of the MPs, as was observed (Fig. 2B). FTIR spectrum from MPs before conjugation to peptides showed a transmittance peak around 2071 cm^−1^, which is attributed to the asymmetric stretching vibration of the free azide groups (Fig. 2C. a). The free azide group was absent in the spectra of peptide-conjugated MP samples due to the conversion of free azide groups to triazole ring during azide-alkyne cycloaddition (Fig. 2C. b, c). The peak observed at 1647 cm^−1^ in p-IPL could represent carbonyl groups of amide bonds of the peptide (Fig. 2C. b). The bands at wavelengths between 1400–1650 cm^−1^ correspond to aromatic rings found on p-LPS. (Fig. 2C. c).

**Fig. 3:**
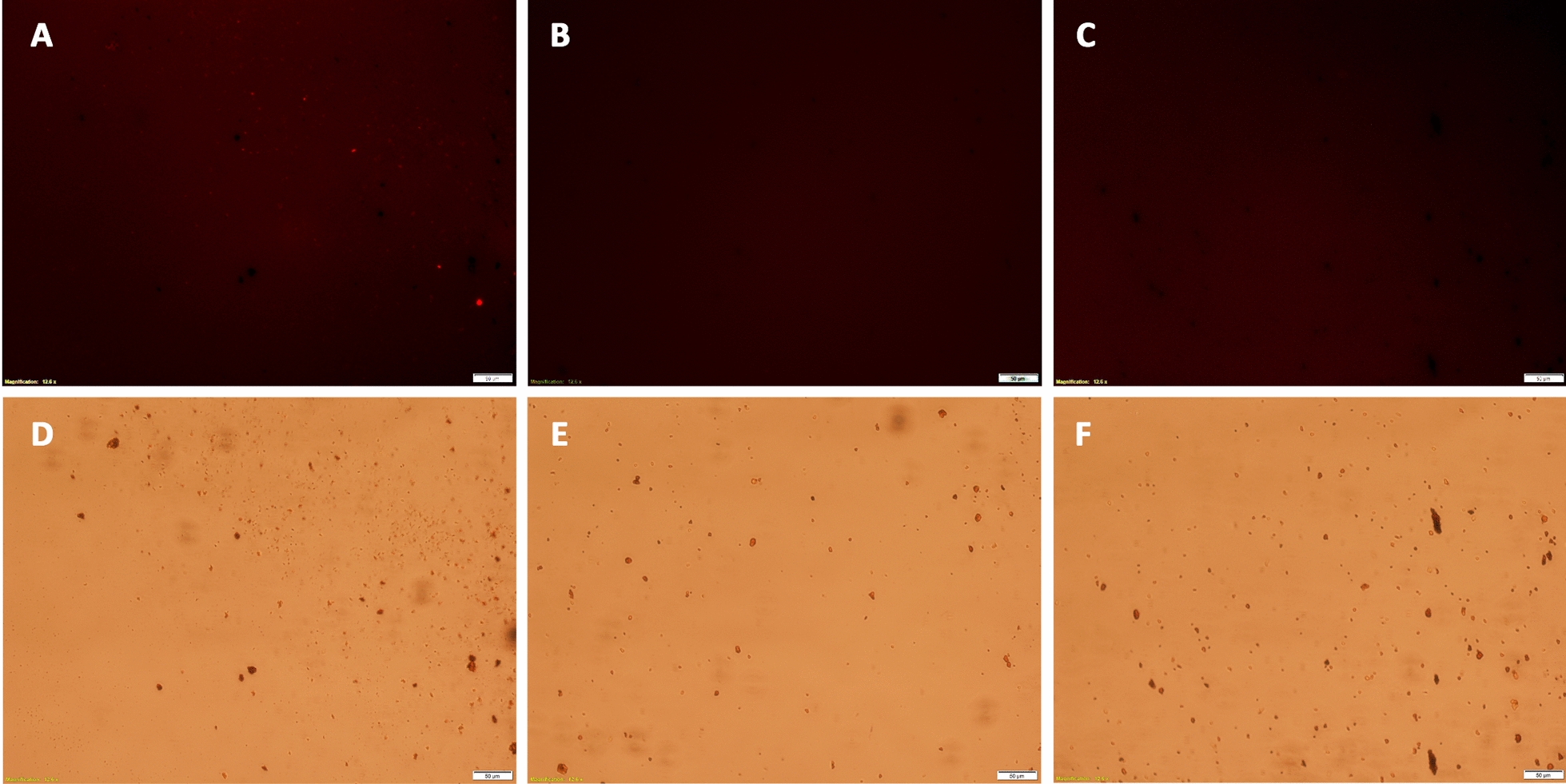
Competitive labeling of MPs with TAMRA dye. Peptide-conjugated MPs and azide-functionalized MPs without conjugated peptides were labeled with TAMRA dye. (**A**, **D**) Control particle clumps having free azide groups showed noticeably higher fluorescence under the microscope. MPs conjugated to p-IPL (**B**, **E**) and p-LPS (**C**, **F**) showed no fluorescence under the microscope. Top images were taken in the fluorescence mode using TRITC filter and bottom images were taken in the bright field mode to confirm the presence of particles and clumps of particles. (Scale bars = 50 µm)

Conjugation of peptides to MPs was confirmed by labeling particles with or without conjugated peptides with an azide-reactive dye (TAMRA alkyne fluorophore), where subsequent fluorescence indicated that unreacted azides were present on the MP surface (Fig. 3). MPs were reacted with p-IPL (Fig. 3B) or p-LPS peptides (Fig. 3C), those MPs had no fluorescence compared to the control MPs that had no conjugated peptides (Fig. 3). Given the excessive amount of peptides used to react with available azides on the magnetic particles, coupled with the lack of any unreacted azides (Fig. 3B, C), it is reasonable to conclude that nearly 100% of all azides reacted to covalently tether peptides to the magnetic particles.’

### Targeting capability of MP-peptide conjugates

**Fig. 4:**
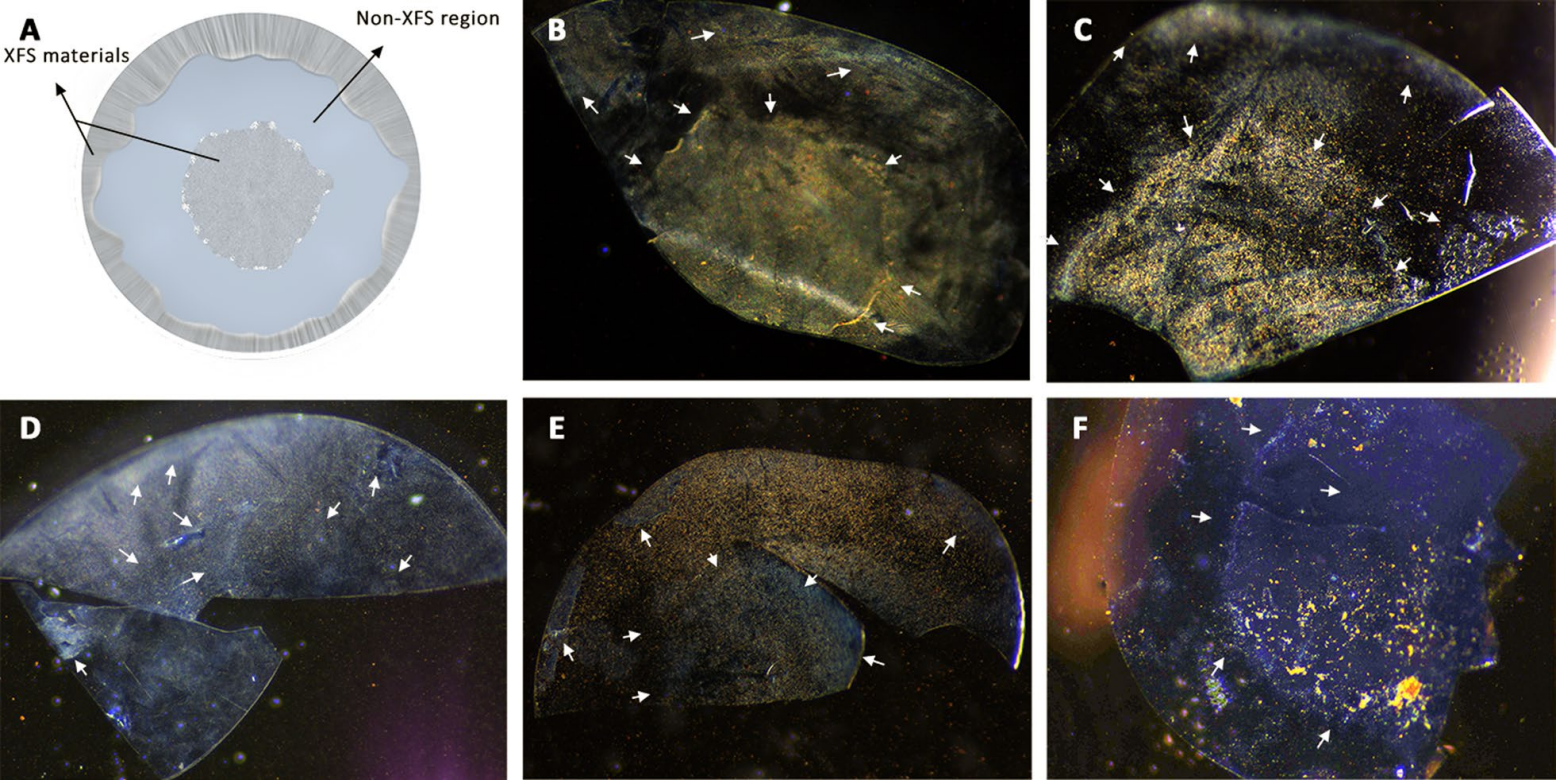
Targeting of XFS materials on the surface of the human lens capsule with MP-peptide conjugates. (**A**) Illustration of the anterior lens capsule showing general pattern of XFS deposits on its surface. (**B**) MP-p-IPL showed specific binding to XFS deposits on both central and the peripheral zones of tissue having XFS materials. The non-XFS area of lens capsule (blank spots) showed less or no particles compared to the exfoliated area. (**C**) MP-p-LPS also showed specific targeting of XFS materials on the surface of the lens capsule. (**D**) Scrambled MP-p-IPL interacted non-specifically with both XFS deposits and the area of lens capsule surface without XFS deposits. (**E**) Scrambled MP-p-LPS also showed non-specific interaction with XFS materials. (**F**) MP without peptide conjugates. As shown in the image, there is a high amount of XFS materials on the central part of the human lens capsule, however, there is not enough binding of MPs to the XFS area. Arrows indicate approximate regions of XFS materials in each tissue sample

It is important to note that although there might be patient-to-patient variations in the XFS deposition pattern on the lens capsule, generally it is distributed with a non-XFS intermediate zone that separates exfoliative central and peripheral zones (Fig. 4A) [11]. Specific targeting of XFS materials was evaluated through incubating MP-p-IPL, MP-p-LPS, MP-scrambled peptides (control), or unmodified MP (control) with XFS affected lens capsules in equal volume of extracted aqueous humor fluid and BSS irrigating solution at 37 °C. Specific binding of MP-p-IPL and MP-p-LPS to XFS materials was observed (Fig. 4B, C). This confirms that MP-peptide conjugates resulted in similar binding patterns as that observed for just fluorescently labeled phage-displayed peptides (Fig. 1). Whereas, scrambled peptide complexes showed a non-specific binding and the whole surface of the lens capsule having was covered with MPs regardless of XFS presence (Fig. 4D, E). The other control experiment using virgin MPs resulted in large clumps of MPs on the central zone (Fig. 4F).

### Effect of magnetic field on XFS materials

Micron-sized, iron oxide particles were used as they are the only metal oxide particle clinically approved for biomedical applications, less susceptible to nonspecific cellular uptake or vascular egress, rapidly cleared (< 5 min) by the liver and spleen, and have a high labeling valency that enhances their binding affinity to molecular targets [21, 27–30]. These particles are biodegradable, where particles (> 150 nm) are captured by phagocytic cells and their coating cleaved by lysosomal enzymes, and the iron oxide core is degraded into iron and oxygen through mechanisms involved in iron metabolism [31, 32].

### Magnetic pin test

**Fig. 5:**
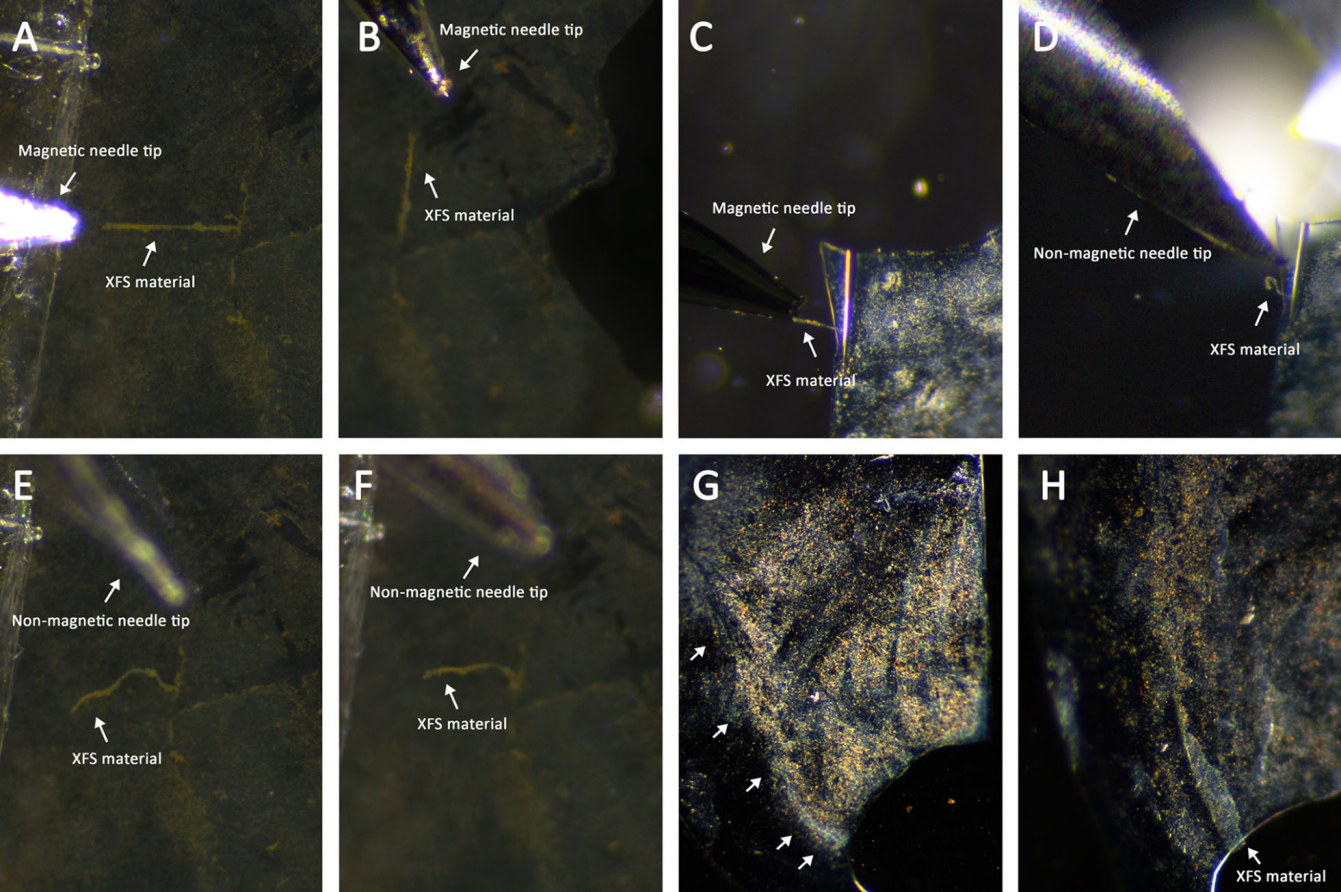
Effect of magnetic pins on the magnetized XFS materials. Human lens capsules having XFS materials were incubated with MP-peptide conjugates and subsequently, magnetic behavior of XFS aggregates was studied using magnetic and non-magnetic tools. (**A**, **B**) Large aggregates of XFS materials covered with MP-p-IPL were pulled by magnetic pin in different directions. (**C**, **D**) XFS materials in the center of lens capsule covered with MP-p-LPS were attracted to the magnetic pin. However, in the absence of a magnetic field (i.e. non-magnetic needle) no attraction was observed. (**E**, **F**) Control studies with non-magnetic needles showed that XFS materials covered with MP-p-IPL did not react to the non-magnetic tool. (**G**) XFS materials in the center of the human lens capsule covered with MP-peptide conjugates before application of magnetic pin. (**H**) Edges of the same XFS deposits in the central zone of the lens capsule being pulled towards the applied magnetic field through a magnetized pin

A magnetized pin was used to prove that MP-bound XFS fibrils could be affected via a localized magnetic field. The magnetic force generated at the tip of the pin was not strong enough to remove MP-XFS aggregates, it was able to re-orient them in the direction of the applied field (Fig. 5A–C); contrary to a non-magnetic needle control (Fig. 5D–F). For some lens capsules these magnetic pins did start to pull the edges of XFS materials off the lens capsule, but was unable to remove these deposits entirely (Fig. 5G, H). However, any XFS materials that were already detached and in solution due to irrigation or surgical manipulation could be collected using a magnetized pin.

### Rotating magnetic field studies

Using the same experimental strategy, a rotating magnetic field was employed to liberate XFS materials from the lens capsule. It has been shown that the oscillation of disk-shaped MPs, via low-frequency rotating magnetic fields that produced a uniform 10,000 G magnetic field, could generate enough destructive force to induce cancer cell death [21]. In this work, ex vivo studies were conducted using spherical MPs in combination with a 5000 G uniform magnetic field to generate mechanical force for the removal of XFS deposits. Magnetic field-treated tissues were then irrigated with BSS buffer to observe any possible aid in further removal of XFS materials from the tissues.

**Fig. 6:**
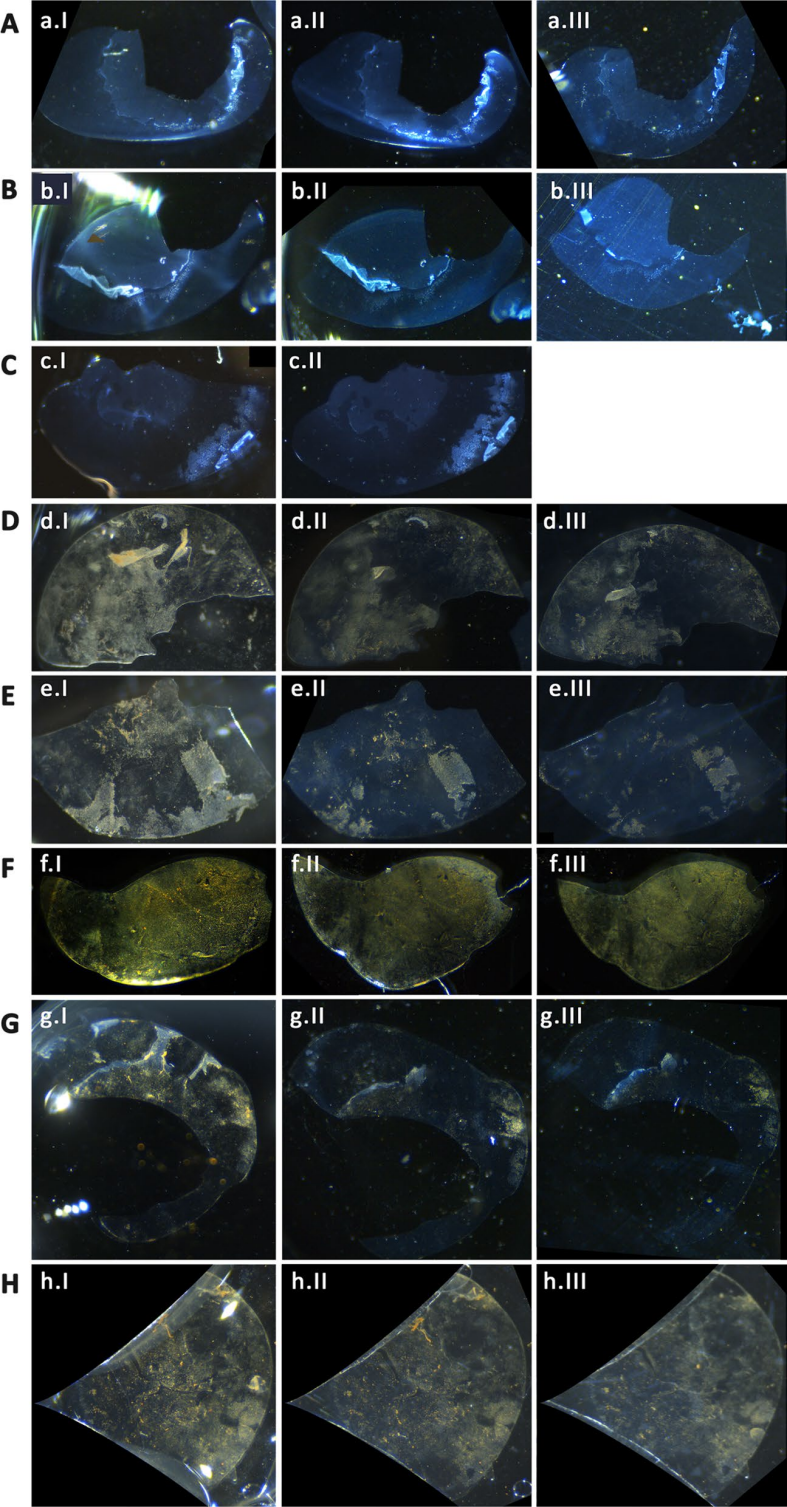
Effect of rotating magnetic field on XFS materials. (**A**–**C**) Control XFS lens capsules treated with rotating magnetic field without MPs. (**D**–**F**) XFS lens capsules interacted with MP-p-IPL. (**G**, **H**) XFS lens capsules interacted with MP-p-LPS. In each raw, images numbered as “I” represent tissues before treated with rotating magnetic field, images numbered as “II” represent XFS lens capsule after 3 h treatment with rotating magnetic field, and images numbered as “III” represent lens capsules after buffer irrigation over the tissue

A rotating Halbach array was used to induce an external magnetic field on XFS laden lens capsules incubated with peptide-decorated MPs. It was observed that the field strength generated was sufficient enough to lead to the removal of a significant amount of XFS materials from the surface of the lens capsule (Fig. 6). Furthermore, the effect of irrigation after agitation by MPs under the magnetic field was used to evaluate the removal of these materials. It was observed that irrigation after magnetic field treatment lead to a further significant removal of XFS materials as compared to irrigation or MP treatment alone. As an example, a lens capsule with central zone deposits that was bound to MP-p-IPL particles (Fig. 6D) showed a large amount of XFS removal upon applying the rotating magnetic field (Fig. 6DII). Another tissue having XFS deposits on both central and peripheral zones (Fig. 6E), bound with MP-p-IPL particles, showed that the application of the rotating magnetic field removed aggregates from both zones of the tissue (Fig. 6EII). MP-p-IPL bound materials on a lens capsule with XFS deposits on the central zone had all large XFS aggregates removed only through the magnetic field (Fig. 6F). However, this tissue had a dense amount of cataractous materials on the posterior side of the lens capsule, which caused the attachment of MPs to those materials making the evaluation difficult. Therefore, although not as visually effective as the previous tissues, the rotating magnetic field was still actually effective in the removal of the large XFS aggregates from the surface of that lens capsule (Fig. 6FII). Lens capsules shown in (Fig. 6G, H)were exposed to MP-p-LPS conjugates and treated with the rotating magnetic field. The central zone of one sample (Fig. 6G) was lost due to surgery, however, XFS materials on the lens surface were mostly removed after applying the rotating magnetic field (Fig. 6GII). The rotating field was also effective in removing some of XFS aggregates from the other tissue (Fig. 6H) incubated with MP-p-LPS. In this case, the peripheral zone of the tissue was mostly cut out of the images due to the coverslips used for holding the tissue in place during irrigation. Irrigation of BSS buffer over the tissue was shown to be effective in removing some XFS aggregates from the surface of the lens capsule (Fig. 6D–H). The control tissues that were treated with the rotating magnetic field showed almost no removal of XFS materials (Fig. 6A–C). Due to the tissue handing difficulties, we could not do BSS irrigation on one of the control tissues (Fig. 6C). XFS materials being partially lost in the control lens capsule shown in (Fig. 6BIII) was not due to the effect of magnetic field, but rather to sample handing being responsible for the loss of the fragile materials that were already lifted from the lens capsule. The control lens capsule shown in (Fig. 6A) showed a very little removal of XFS aggregates during BSS irrigation and no effect was observed after treating with rotating magnetic field (Fig. 6AIII).

**Fig. 7:**
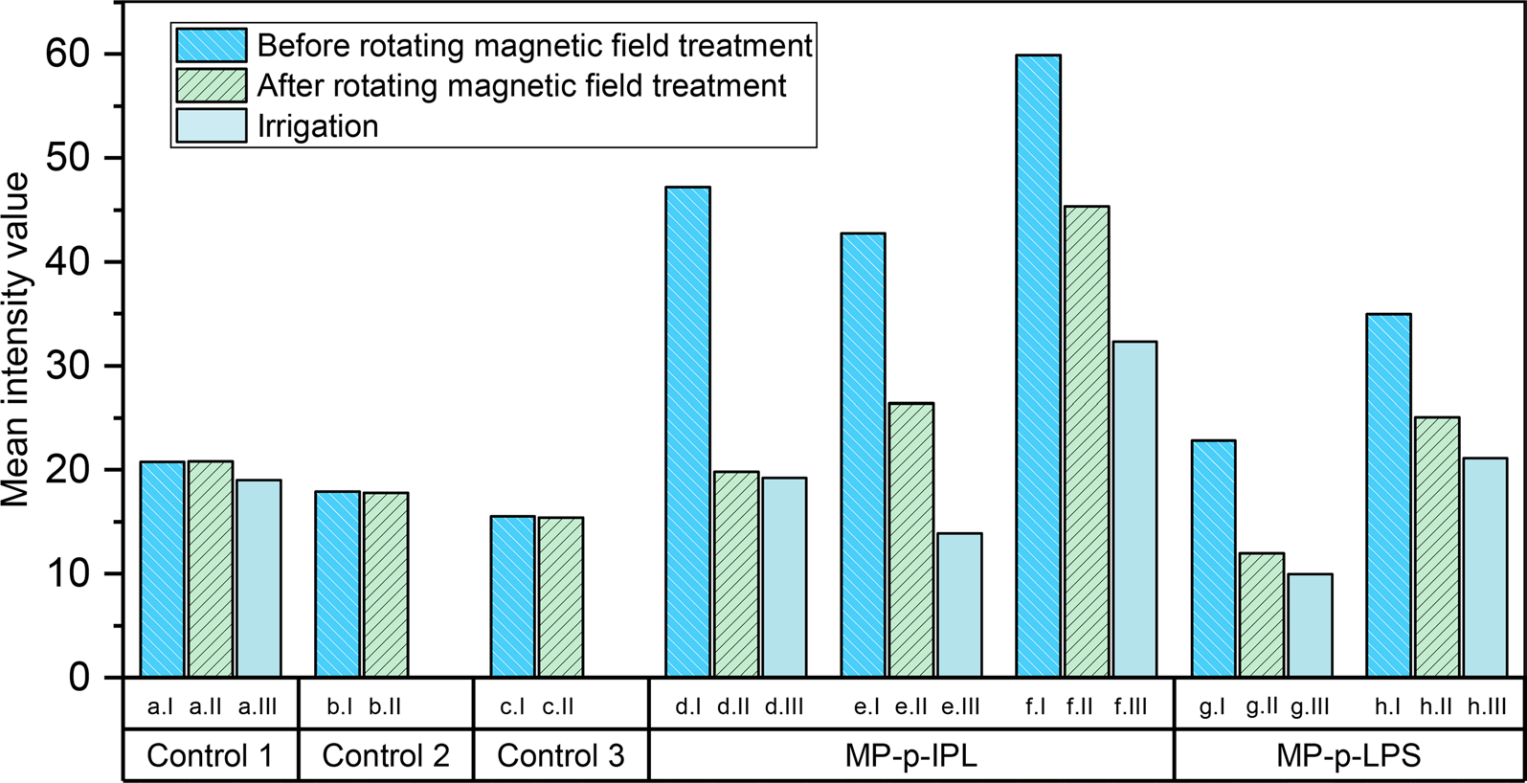
Mean intensity measurements of XFS lens capsules incubated with MP-peptide conjugates. (Control 1-Control 3) Control tissues incubated with BSS and treated with rotating magnetic field. (MP-p-IPL) Representative mean intensity values measured using images of XFS lens capsules interacted with MP-p-IPL. (MP-p-LPS) Mean intensity values of different lens capsules interacted with MP-p-LPS. Both groups of graphs related to test samples showed that intensities were decreased due to the removal of XFS materials from the surface of lens capsules. In each graph, column “I” represents intensity value of the images of the surface of lens capsule before treating with rotating magnetic field, column “II” represents values after 3 h treatment with the rotating magnet, and column “III” represents intensities after applying BSS irrigation over the control XFS lens capsules. In all images, intensity values have been reported after background subtraction

To better evaluate the effectiveness of the observed rotating magnetic field, images of tissues before and after applied magnetic field and irrigation (Fig. 6), were analyzed based on the intensity of the lens capsule surface before and after each test (Fig. 7).

### Cytotoxicity studies of MP-peptide conjugates

Prior to toxicity studies, the common phenotypic features of cultured hTM cells were assessed using immunohistochemical studies (Additional file 1: Fig. S1). The in vitro cytotoxicity of peptides in free and MP-conjugated form was evaluated against obtained hTM cells using live/dead cell viability assay, MTT assay, and DNA cleavage. All in vitro cell assays showed that both free peptide and MP-peptide particles were not toxic in the concentrations studied, as detailed below.

### Live/dead cell viability assay performed on hTM monolayers

**Fig. 8:**
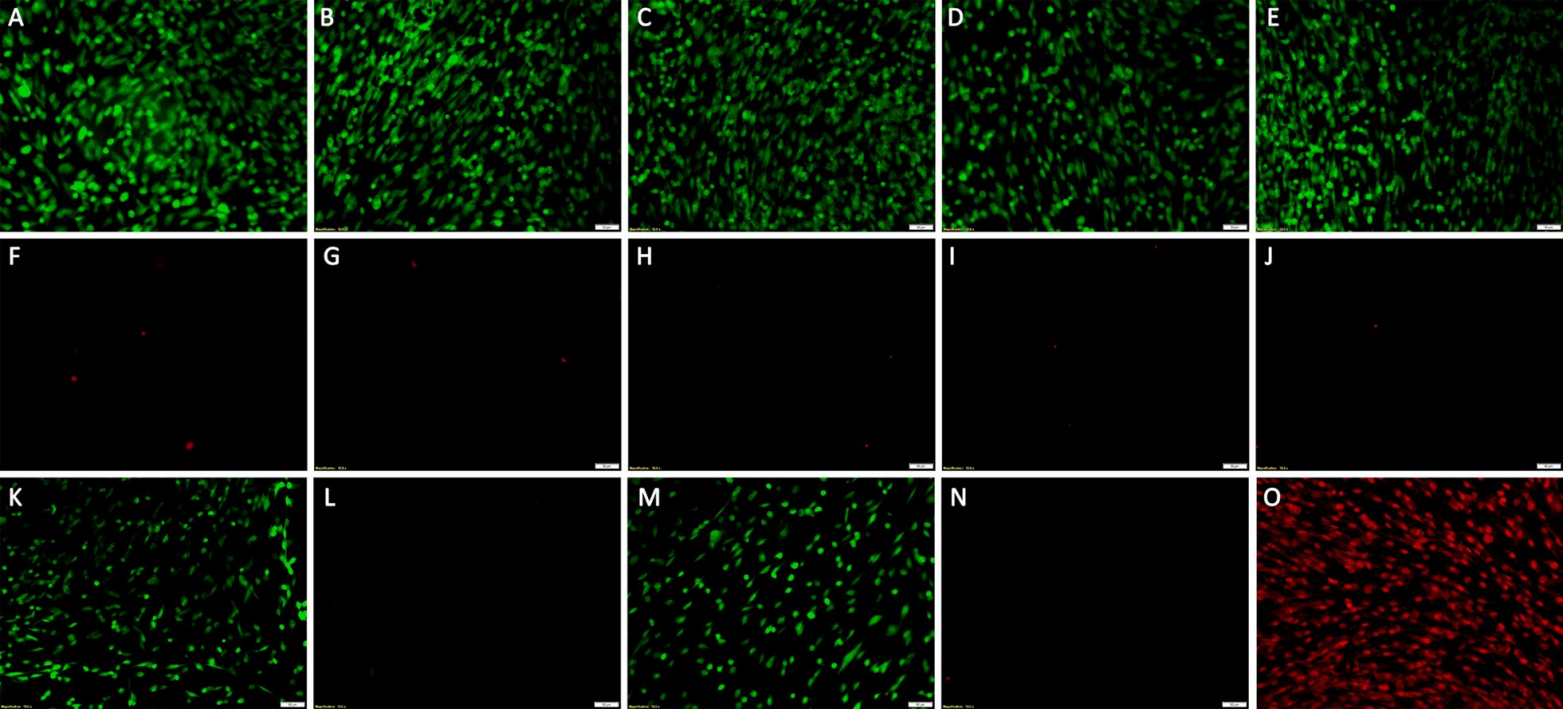
Cell viability (live/dead) assay of hTM cells incubated with free peptide and MP-peptide constructs. Calcein AM (CAM)/ EthD-1 system: Calcein AM detects cellular esterase activity of live cells, EthD-1 stains nuclei of dead cells. The green cells are live (CAM) and red cells are dead cells (EthD-1). (**A**, **F**) Control cells treated with water (without MPs). (**B**, **G**) hTM cells incubated with 50 µg MP-p-IPL for 24 h. (**C**, **H**) hTM cells incubated with 100 µg MP-p-IPL for 24 h. (**D**, **I**) hTM cells incubated with 50 µg MP-p-LPS for 24 h. (**E**, **J**) hTM cells incubated with 100 µg MP-p-IPL for 24 h. (**K**, **L**) hTM cells treated with 1 mM free p-IPL peptide solution (without MPs). (**M**, **N**) hTM cells treated with 1 mM free p-LPS peptide solution (without MPs). (**O**) All cells dead (methanol treated). Each treatment included three biological and three technical replicates. (Scales bars = 50 µm)

XFS-specific peptides in both solution free or MP bound form were incubated with hTM cells and their cytotoxic effects assessed. It was found that both MP-p-IPL and MP-p-LPS constructs were not toxic in the concentrations studied. Green represents live cells and red represents dead cells in (Fig. 8). Confluent cultured cells were incubated with 50 µg and 100 µg of MP-p-IPL or MP-p-LPS (Fig. 8B–E). hTM monolayers were also incubated with 1 mM of p-IPL or p-LPS peptide solutions, and their viability was analyzed in a similar way as the MP-peptide constructs. As seen in the case of MP-peptide conjugates, free peptides were not toxic in used concentrations (Fig. 8K, N) and Table 1). Live/dead assay of hTM cells treated with rotating magnetic field also showed that cells incubated with MP-peptide conjugates did not show significant loss compared to control cells incubated with water (Table 2). Because of the sensitivity of hTM cells, it was not possible to treat cells with a rotating magnetic field outside of a CO_2_ regulated atmosphere for longer than 10 min. That said, even control cells showed significant loss upon removal from the CO_2_ incubator, thus, an optimal 10 min exposure to the magnetic field was chosen to evaluate the viability of cells in the presence of rotating magnetic field (Table 2).

**Table 1:** Live/dead assay performed on hTM monolayers treated with MP-peptide conjugates and free peptide solution. No statistical significant difference (P < 0.05) was found between studied groups.

Peptide-particle conjugates		Free peptide solution	
Sample	% of live cells	Sample	% of live cells
Control	99.01 ± 0.51	Control	99.36 ± 0.36
MP-p-ILP (50 µg)	99.60 ± 0.18	1 mM p-IPL	99.00 ± 0.37
MP-p-ILP (100 µg)	99.66 ± 0.07	1 mM p-LPS	99.12 ± 0.94
MP-p-LPS (50 µg)	99.58 ± 0.20		
MP-p-LPS (100 µg)	98.47 ± 1.46		

Data represent mean ± 1 SD, n ≥ 3

**Table 2:** Live/dead assay performed on hTM monolayers incubated with MP-peptide conjugates and treated under 10 min rotating magnetic field. No statistical significant difference (P < 0.05) was found between studied groups.

Peptide-particle conjugates	
Sample	% of live cells
Control	98.74 ± 0.45
MP-p-ILP (100 µg)	98.53 ± 1.10
MP-p-LPS (100 µg)	98.88 ± 0.51

Data represent mean ± 1 SD, n ≥ 3

### MTT cell proliferation assay

**Fig. 9:**
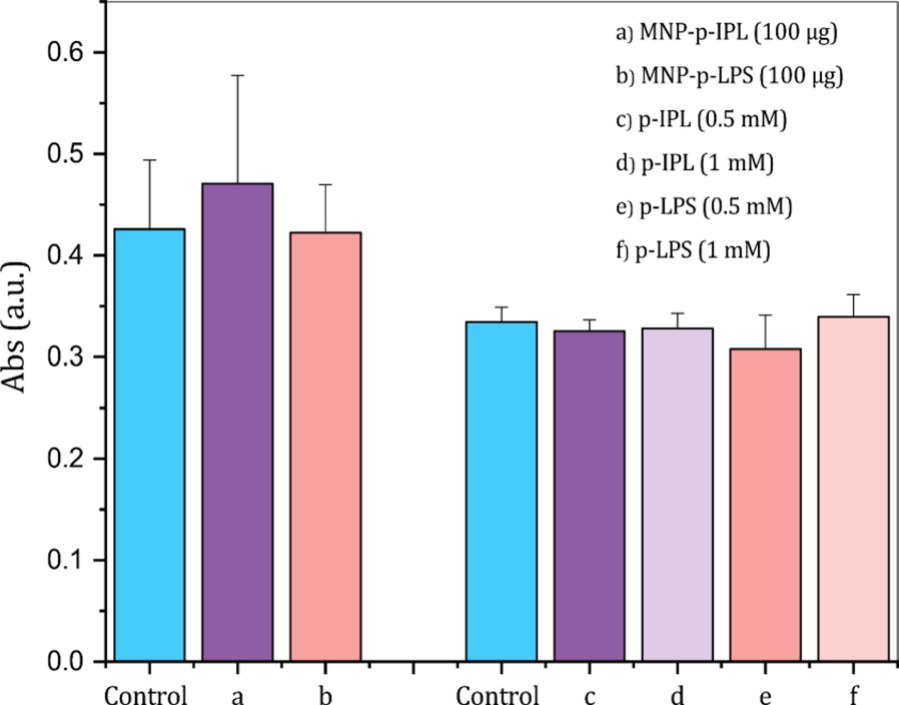
MTT colorimetric assay of viable hTM cells in the presence of XFS-specific peptides in free and conjugated form. (Left) MTT assay conducted using peptide-conjugated MPs. (Right) MTT assay results after incubation of hTM cells for 24 h with 0.5 mM and 1 mM free peptide solutions. The optical density was measured at 570 nm after 24 h incubation of hTM cells with each test sample. (Data represent mean ± 1 SD, n ≥ 3, no statistical difference was observed between the means)

hTM cell proliferation in the presence of solution free and MP bound peptides was conducted using the MTT colorimetric assay. A one-way ANOVA test showed that no significant difference (p < 0.05) was observed between the absorbance of hTM cells treated with MP-peptide conjugates or solution free peptides compared to control cells treated with water (Fig. 9). The results confirmed that both sets of test samples did not suppress the proliferation of hTM cells.

### DNA fragmentation

**Fig. 10:**
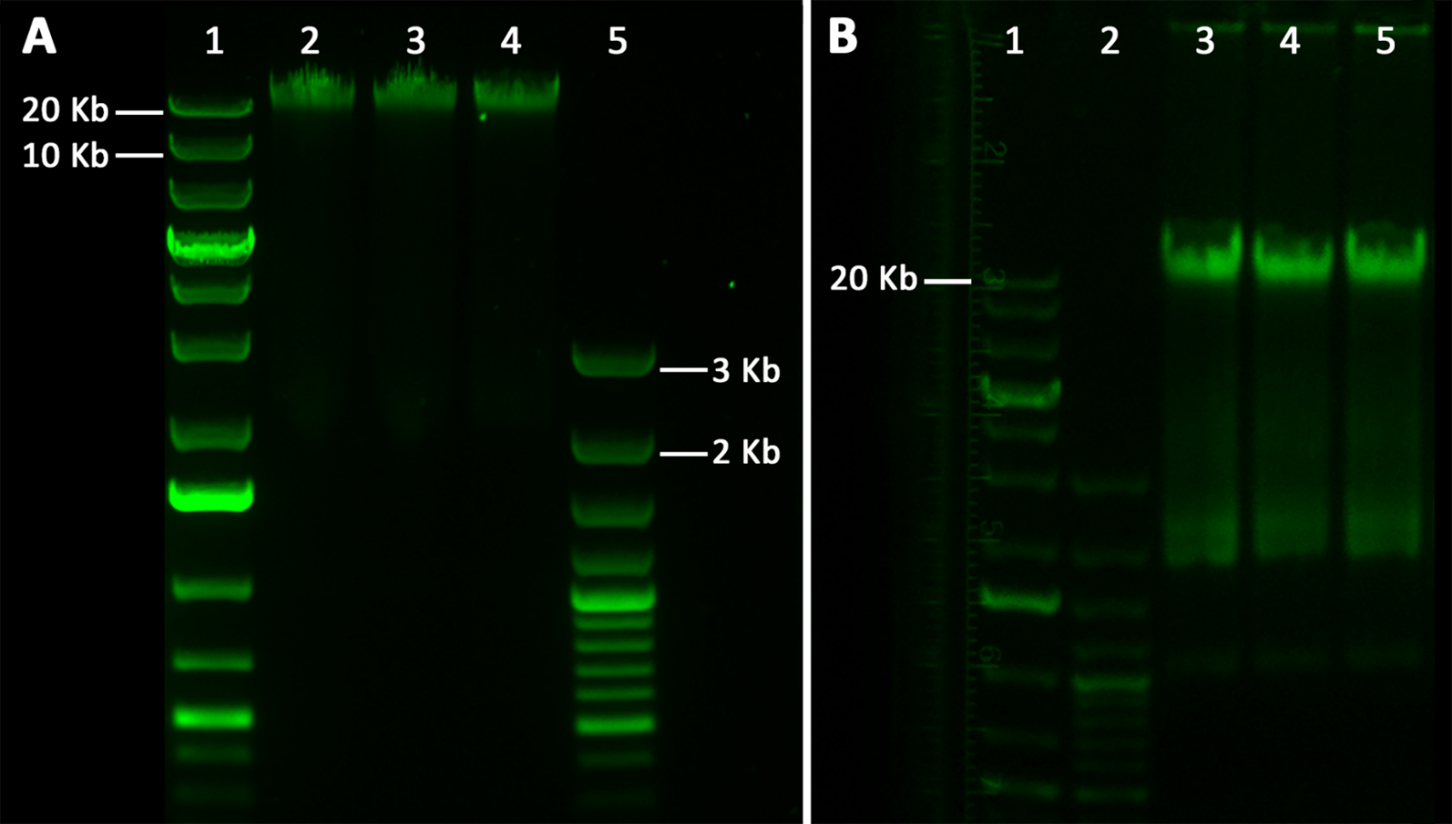
Electrophoretic analyses of the apoptotic chromosomal DNA fragmentation of hTM cells. (**A**) Isolated DNA from hTM cells after being incubated with MP-peptide conjugates for 24 h. lane 1: 1 Kb plus DNA ladder, lane 2: Control, hTM cells treated with water, lane 3: Extracted DNA of hTM cells incubated with 100 µg MP-p-IPL for 24 h, lane 4: Extracted DNA of hTM cells incubated with 100 µg MP-p-LPS for 24 h, lane 5: 100 bp plus DNA ladder. (**B**) Isolated DNA from hTM cells incubated with free XFS-targeting peptide solutions. Lane 1: 1 Kb plus DNA ladder, lane 2: 100 bp plus DNA ladder, lane 3: Control, hTM cells treated with water, lane 4: hTM cells incubated with 1 mM MP-p-IPL for 24 h, lane 4: hTM cells incubated with 1 mM MP-p-LPS for 24 h

Effect of free and conjugated XFS-targeting peptides on the induction of apoptosis was investigated via chromosomal DNA fragmentation analysis: DNA fragments indicate apoptosis induction. MP-peptide constructs, as well as solution free peptides, did not initiate apoptosis for hTM cells; chromosomal DNA remained intact after 24 h incubation with MP-p-IPL, MP-p-LPS, p-LPS, or p-IPL solutions (Fig. 10).

### Cell uptake studies using electron microscopy

**Fig. 11:**
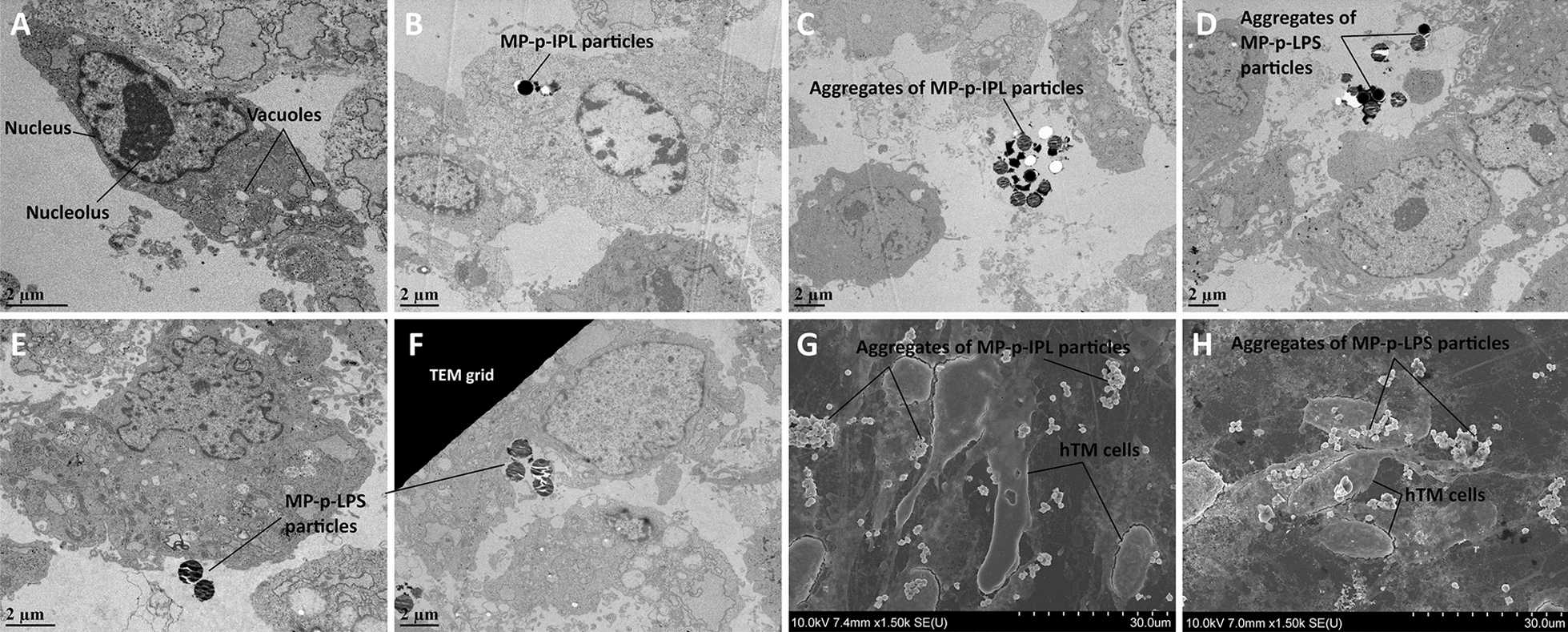
Electron micrographs of hTM cells incubated with MP-peptide conjugates. (**A**–**F)** TEM micrographs. (**G**, **H)** SEM micrographs. hTM cells were incubated with 100 µg of MP- peptide conjugates for 2 h**.** (**A**) Control hTM cells incubated with water. (**B**) MP-p-IPL particles located inside the hTM cells. (**C**) Aggregates of MP-p-IPL particles located outside of the cells. (**D**, **E**) Aggregates of MP-p-LPS particles located outside of hTM cells. (**F**) MP-p-LPS particles being engulfed by the hTM cell. Black dirt between particles could be trapped dye molecules. (**G**) SEM micrograph showing aggregates of MP-p-IPL particles around hTM cells. (**H**) SEM micrograph of MP-p-LPS particles located around the hTM cells

One of the key functions of trabecular cells is the phagocytosis of foreign materials and extracellular debris [33], a cellular function also observed in vitro [34]. The internalization of MP-peptide constructs into hTM cells may lead to cell death if exposed to an oscillating magnetic field. Thus, hTM cells were incubated for 2 h with 100 µg of each of MP-p-IPL or MP-p-LPS and TEM micrographs showed that individual particles were taken up by hTM cells (Fig. 11B), and some particles were engulfed by hTM cells (Fig. 11E, F). Aggregates of both MP-p-IPL and MP-p-LPS particles were observed outside of cultured hTM cells as well (Fig. 11C, D). Individual particles, as well as aggregated MPs, were also observed in SEM micrographs of regions surrounding cultured hTM cells (Fig. 11G, H). It seems that MPs in large aggregates have less propensity to enter the cultured cells. It has been previously shown that intravitreal or anterior chamber injection of magnetic nano- and microparticles had almost no signs of effect on retinal morphology, photoreceptor function or IOP in the anterior chamber of animal models [35]. Regardless, according to different cell viability assays conducted in this study, no statistical difference was observed for cell mortality upon incubation with MPs in cultured hTM cells.

## Conclusions

This study set out to identify small peptides having a selective affinity to XFS materials in the human eye. An ex vivo panning procedure was developed to explore targeting peptides for the XFS materials using the phage display technique. The selective affinity of phage-displayed peptides was confirmed through ex vivo studies using human lens capsule and fluorescently labeled phages. XFS-targeting peptides were successfully conjugated to MPs using azide-alkyne cycloaddition click chemistry. FTIR analysis and zeta potential measurements were used to confirm the conjugation of peptides to MPs. Competitive labeling of MPs using alkyne-modified fluorophore was also used to confirm the attachment of peptides to the particles. Targeting capability of MP-peptide complexes to XFS materials was studied ex vivo in the same experimental conditions that phage panning was conducted. Compared to the scrambled peptide sequences, the identified XFS-targeting peptide conjugates showed selective and high affinity to XFS materials on the human lens capsule. Although XFS materials have been clinically characterized with a general deposition pattern on the lens capsule, there are variations from patient to patient. Considering those variations, results of the ex vivo experiments showed that the identified peptides had acceptable selective binding to XFS materials in most of the lens capsule areas associated with XFS materials. The effect of the magnetic pin and rotating magnetic field on the behavior of magnetized XFS materials was observed under the stereomicroscope. A low frequency rotating magnetic field which was producing a uniform magnetic field across the designed system provided suitable conditions to remove most of the XFS aggregates from the surface of lens capsules. Previous work as far back as 2006 has shown that compared to non-XFS cases, XFS patients show greater IOP lowering effect following phacoemulsification cataract extraction, where this IOP decrease was found to be proportional to irrigation volume used during cataract surgery [36]. This has been speculated that washing out of XFS materials could be one of the reasons for observing greater IOP drop in XFS patients [36, 37]. In this study we found that irrigation after MP treatment leads to enhanced removal of XFS materials from the surface of ex vivo lens capsules. Upon removal of XFS materials, the commonly used irrigation/aspiration system can remove large and small XFS materials from the anterior chamber of the eye. It is thought that due to the targeting ability of our designed MP-peptide system against XFS materials, this technique has the potential to eliminate XFS materials from the majority of surfaces in the anterior ocular chamber.

Biocompatibility of free peptides and corresponding MP conjugates was confirmed using MTT cell toxicity assay and live/dead cell proliferation assay. DNA fragmentation studies also showed that either MP-peptide conjugates or free peptide solutions did not induce apoptotic cellular death in hTM cells. Electron microscopy studies showed that compared to the particles being in aggregated form, individual particles were more likely to be taken up by the cultured hTM cells. Considering targeting evaluation findings and biocompatibility studies results, MP-peptide conjugates having specific affinity to XFS materials could provide an innovative tool in the development of a therapeutic approach for XFS whereby removal of large deposits of XFS aggregates from the anterior chamber of affected eyes might help prevent or manage exfoliation related glaucoma. Since the critical buildup of XFS materials may occur over years and that this is a minimally invasive approach for targeting and removal of XFS materials, multiple treatments would not be a burden to either the patient or the clinician relative to the risks associated with current treatments.

Over the past decades, iron oxide magnetic particles have shown promising potential in the development of effective therapeutic agents for many human diseases. In fact, the only approved metallic nanoparticles for clinical use are iron oxide nanoparticles, where there are FDA-approved applications of them for hyperthermia, iron deficiency anemia, and cancer diagnosis [38, 39]. This could also suggest the possible clinical use of this system in the future, however further studies addressing possible risk factors with high concentrations of particles should be conducted [32].

## Methods

### Patient sample collection

Human lens capsules were collected from patients having an age range of 63–84 years, mean 74.8 ± 5 years, undergoing phacoemulsification cataract surgery, and was stored in the balanced salt solution (BSS^®^ intraocular irrigating solution, Alcon) at 4 °C prior to use. Aqueous humor fluid was collected from the anterior chamber of the eye using a 30-gauge cannula inserted through the paracentesis site. Patients with a history of diabetes mellitus, with previous severe trauma to the eye, with previous expositor to infrared radiation and patents with the previous diagnosis of amyloid disease were excluded from the study.

### Cell line

Primary human trabecular meshwork (hTM) cells were obtained from ScienCell Research Laboratories (Carlsbad, CA), and maintained in TMCM medium (ScienCell, no. 6591). Primary cell culture was passaged according to the manufacturer's instructions. Passage three cells were seeded on tissue culture plates coated with gelatin and media were refreshed every 2–3 days. These monolayer cultures were used in subsequent experiments upon reaching 95–100% confluency.

### Isolation of XFS material-specific peptides

**Fig. 12:**
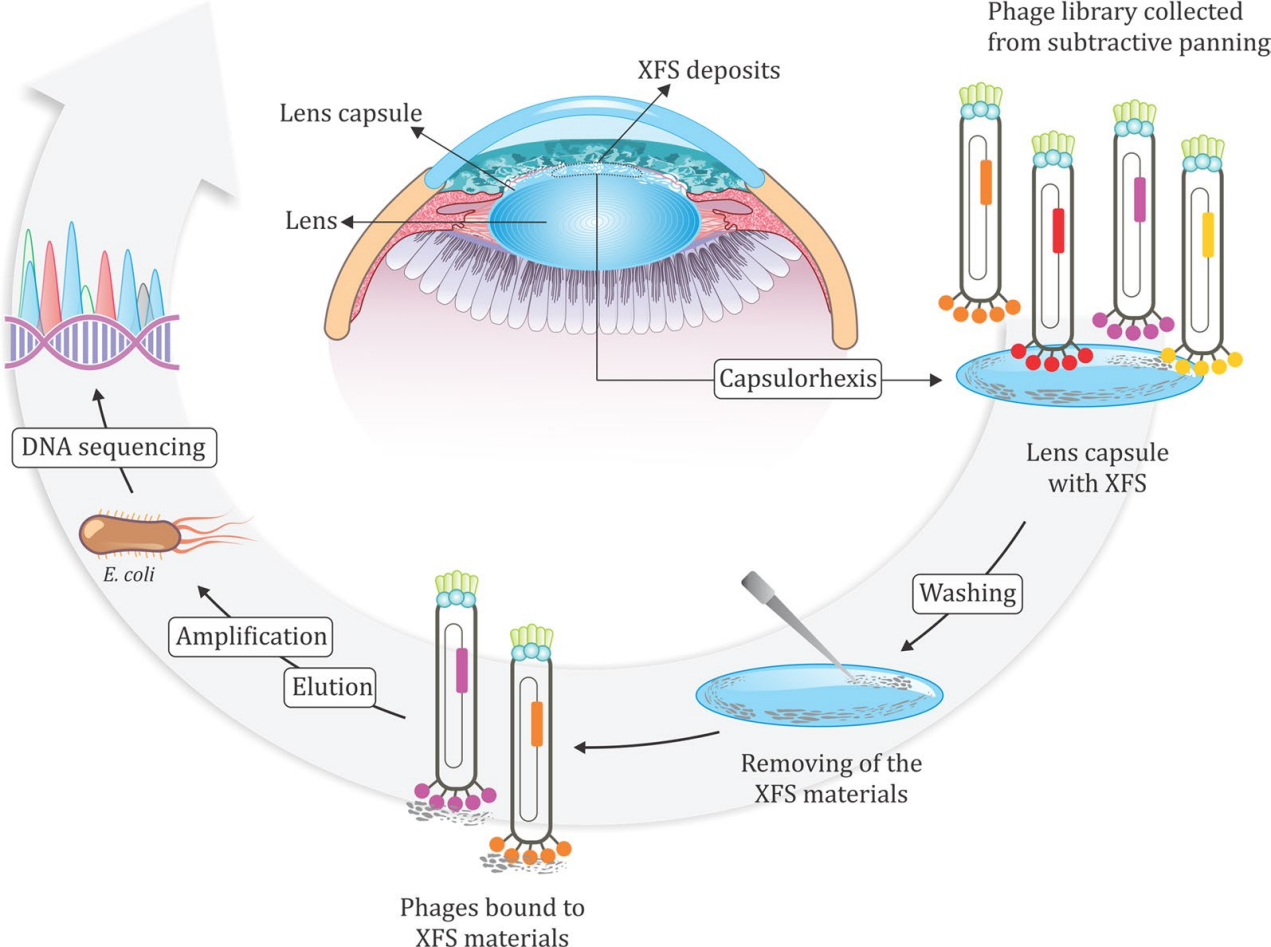
Ex vivo panning of phage-displayed peptide library on exfoliative human lens capsule. Control human lens capsules without XFS materials were subjected to subtractive panning to enrich XFS materials-specific binders. Positive screening was carried out on lens capsules collected from patients with XFS and undergoing phacoemulsification cataract surgery. Three rounds of screening were applied and selected phage plaques were analyzed using DNA sequencing

Ph.D.^™^-12 phage display peptide library was used for ex vivo screening. All human tissues were washed three times with BSS buffer before use. Human lens capsules collected from patients without XFS were used for subtractive screening. The lens capsules were incubated with the phage library (1 . 10^11^ pfu) in an equal volume of aqueous humor fluid and BSS® irrigating solution for 1 h at 37 °C in 0.2 ml tube. The solution was removed and 300 µl of ice-cold BSS solution was added to the tube and tissue was washed several times with BSST buffer (BSS solution containing 0.1% v/v Tween-20) to elute off unbound or weakly bound phages. The lens capsule was subsequently stained with 0.06% trypan blue (same concentration used in anterior segment surgeries to facilitate visualization of target tissues in specific situations) to have a better visualization of XFS materials under the microscope. Tissue was washed further to remove the excess dye and was placed on a sterile microscope slide and covered with 20 µl of BSS buffer and XFS materials were carefully removed from the surface of the lens capsule using gel-loading pipette tips (GELoader, epT.IPS, 20 μL, Eppendorf, Germany). Care was taken to not remove undesired parts of the lens capsule during the process. The solution containing the isolated XFS materials was transferred to a fresh tube containing 100 µl 0.2 M glycine–HCl (pH 2.2) to elute phages bound to the XFS materials. After 15 min the solution containing recovered phages was neutralized with 15 µl 1 M Tris–HCl buffer (pH 9.1). The eluted phages were amplified by infection of *E. coli* host strain ER2738 (New England Biolabs). Three rounds of ex vivo panning were carried out with stepwise increasing of Tween concentration in BSST buffer (0.1, 0.2, 0.3%) to increase the likelihood of identification of XFS materials-targeting peptides. Individual clones were then picked for the characterization of peptide-encoding inserts using DNA sequencing. 12 clones were picked from the first and second round each and analyzed via DNA sequencing to make sure that there was no growth advantage happening over the library at the beginning of the screening. 52 clones were subsequently picked from the ex vivo*-*identified XFS-targeting phages and their peptide-encoding DNA inserts were analyzed using DNA sequencing (Fig. 12).

### Evaluation of the targeting ability of phage-displayed peptides

#### Phage labeling

The ability of two highly enriched phage-displayed peptides to bind specifically to XFS materials was evaluated ex vivo by fluorescently labeling these phages with Cy5 (Lumiprobe) as described elsewhere [40]. Amplified phages (1 × 10^11^ pfu) were resuspended in 0.3 M NaHCO_3_ (pH 8.6) containing 10 µg Cy5 dye and incubated for 2 h at room temperature in the dark. Subsequent to phage/fluorophore incubation, 40 µl of 10 mM lysine was added to interact with remaining free Cy5 dye molecules in the solution. The volume of the reaction mixture was subsequently brought up to 1 ml with PBS buffer, and the phages were purified with two rounds of 20% (w/v) polyethylene glycol-8000, 2.5 M NaCl precipitation. The labeled phages resuspended in BSS solution.

### Ex vivo evaluation of targeting ability of labeled phages

Fluorescently labeled phages carrying identified targeting peptides as well as wild-type phages without peptide-encoding inserts were incubated with exfoliative human lens capsules in a 100-µl solution containing equal volumes of human aqueous humor fluid and BSS solution. The incubation was allowed to continue for 1 h at 37 °C. After serially washing with BSST buffer (0.1, 0.3%), three times each, the lens capsules were mounted on microscope slides and were examined under an Olympus IX81 inverted fluorescence microscope (Olympus Corporation, Tokyo, Japan). The location of XFS materials on the surface of the lens capsule was confirmed in bright-field mode prior to fluorescence imaging.

### Magnetic bead-peptide conjugates

Azide-functionalized iron oxide core magnetic particles with biocompatible coatings and having a diameter of 1 μm (purchased from Nanocs Inc, New York) were used in this study. Synthetic alkyne-modified peptides (≥ 95% purity) corresponding to the phage-displayed XFS materials-binding peptides and scrambled sequences were purchased from RS synthesis (Louisville, KY, USA). The peptides were alkyne modified and their conjugation to azide-functionalized MPs were carried out through copper-catalyzed azide-alkyne click chemistry as described Copper-Catalyzed Azide–Alkyne Click Chemistry for Bioconjugation [41]. Azide-functionalized MPs were added to the peptide solution having a final concentration of 600 µM in 100 mM potassium phosphate buffer (pH 7). A premix solution containing 2.5 µl of 20 mM CuSO_4_ and 5 µl of 50 mM tris(3-hydroxypropyltriazolyl-methyl)amine (THPTA) ligand (Lumiprobe) was prepared immediately prior to use and added to the click reaction solution. 25 µl of 100 mM sodium ascorbate was subsequently added and the reaction was allowed to proceed for 1 h. Subsequent to click reaction, the peptide-conjugated MPs were first washed with 10 mM EDTA to remove copper ions and then with BSS buffer.

### Characterization of peptide-conjugated MPs

The surface charge of MPs with and without peptide conjugates was measured using a Zetasizer Nano ZS (Malvern Instruments, UK). For FTIR measurements, drop-cast films of MPs with and without peptide conjugates were analyzed using an FTIR microscope (Nicolet continuum FTIR microscope (Thermo Scientific). FTIR spectra were collected with a resolution of 4 cm^−1^ and 128 scans of each sample. Conjugation of peptides to MPs was further analyzed through competitive labeling of MPs with 5-carboxytetramethylrhodamine alkyne (TAMRA-alkyne), 5-isomer fluorophore (Lumiprobe). Azide-functionalized MPs were conjugated first with targeting peptides and then labeled with TAMRA-alkyne fluorophore as described before in the conjugation section. Since the azide groups on the surface of MPs had already interacted with the alkyne group of peptides, they were expected to be non- (or less-) labeled compared to control particles (without peptide conjugates).

### Ex vivo evaluation of targeting ability of identified peptides

Human lens capsules obtained from XFS patients after washing with BSS buffer were transferred into a solution containing equal volumes of human aqueous humor and BSS irrigating solution. 25 µg of each control MPs (without peptide conjugates), MP-peptide, and scrambled peptide-MP complexes were incubated with lens capsules in that solution in a 0.2 ml tube for 1 h at 37 °C with gentle shaking. Afterward, the excess particles were washed off with BSS buffer and the lens capsules were mounted on the microscope slide and images were taken using an Axiocam-105 color camera on a stereomicroscope (Stemi-305, Carl Zeiss).

### Evaluation of behavior of magnetized XFS materials under magnetic field

#### Ex vivo evaluation using magnetic pin

Human lens capsules incubated with MP-peptide complexes were laid flat on a microscope slide with the XFS side facing up and 50 µl of BSS buffer was placed on the top of the tissues. A metallic pin which was magnetized by attaching it to the surface of a permanent magnet having field strength at the pole of ~ 5000 G was used to observe the effect of applied magnetic tool on the XFS materials. Control experiments were carried out on the same tissues using a non-magnetic 23-gauge needle which had almost the same tip size as previously used magnetic pins. Images were taken under the stereomicroscope.

#### Ex vivo evaluation using rotating magnetic field

The same approach was followed as previously described except for using a rotating magnetic field instead of the static magnetic field applied with a magnetic pin. The lens capsules were first incubated with MP-peptide conjugates and then treated with a rotating Halbach array magnet that produced a ~ 5000 G magnetic field across the gap of the magnet (Nickel-plated N48H, Super Magnet Man Inc., Alabama, USA). The tissue samples were incubated at the entrance of the Halbach array gap rotating at 20 Hz for 3 h and then images were taken under the stereomicroscope. Irrigation and aspiration is used in cataract surgery to remove remaining parts of the lens materials and residual viscoelastic solution from the eye. To mimic the surgery conditions, irrigation of BSS buffer at a rate of 10 ml/min was applied over the processed tissues to observe the effect of buffer irrigation on the removal of the XFS materials after being treated with rotating magnetic field. Control studies were carried out with the same treatment approaches using XFS lens capsules, except for using BSS buffer instead of MP-peptide conjugates.

In order to better evaluate of the effect of rotating magnetic field on removing of XFS materials from the surface of lens capsules, images of lens capsules which were captured before and after treatment with the rotating Halbach array were converted to 16-bit gray-scale images and their intensities analyzed using Adobe Photoshop CC 2015 software (Adobe Systems Inc, San Jose, CA).

### Electron microscopy studies

#### Scanning electron microscopy (SEM)

The monolayer of hTM cells cultured on gelatin-coated glass coverslips in 24-well plates were incubated with 100 µg of MP-peptide complexes for 2 h at 37 °C. The wells were topped up with the fixative solution (2.5% glutaraldehyde, 4% paraformaldehyde in 0.1 M phosphate buffer, pH 7.4), for 20 min. Upon serial washing with PBS buffer (pH 7.4), samples were dehydrated with graded ethanol gradually by 20% increments for 30 min each until 100% ethanol. Samples were subsequently treated with hexamethyldisilazane (HMDS) and allowed to dry overnight, then mounted on SEM stubs and sputtered with a gold–palladium film. SEM images were obtained using a Zeiss Sigma FE-SEM scanning electron microscope (Carl Zeiss, Inc., Oberkochen, Germany) at the accelerating voltage of 20 kV.

#### Transmission electron microscopy (TEM)

Confluent monolayers of hTM cells cultured in 24-well cell culture plates were incubated with MP-peptide complexes as previously described for SEM studies. After washing with Dulbecco’s PBS buffer, cells were scraped off the wells and transferred into the microcentrifuge tube. Cells were centrifuged at 1000 rpm for 5 min to form a pellet. The supernatant was removed and the cells pellet was incubated in the fixative solution overnight. Upon washing with 0.1 M phosphate buffer (pH 7.4), samples were post-fixed in 1% osmium tetroxide for 1 h. Dehydrated pellets were then embedded in Spurr’s resin and allowed to polymerize at 70 °C overnight. Ultrathin sections were prepared using an ultramicrotome (Reichert-Jung Ultracut-E, Vienna, Austria) with a diamond knife. The ultrathin sections were stained with 4% uranyl acetate solution and imaged using a Philips–FEI, Morgagni-268 transmission electron microscope (Hillsboro, USA) operating at an acceleration voltage of 80 kV.

#### Morphology and phenotype of hTM cells

Before biocompatibility studies, the morphology and phenotypic characteristics of cultured hTM cells were studied. Population doubling time of hTM cells was obtained as described elsewhere [42].

#### Immunohistochemical study

Immunohistochemical assays were employed to identify the expression of characteristic phenotypic markers of trabecular meshwork cells. Confluent monolayers were confirmed using an inverted cell culture microscope (Leica DMi1, Leica Microsystems Inc, Germany). Immunohistochemical labeling was conducted according to the manufacturer’s protocol. Rabbit anti-fibronectin (1:200, ab2413, Abcam), rabbit anti-laminin (1:200, ab11575, Abcam) and rabbit anti-myocilin (1:200, ab41552, Abcam) antibodies were used as fluorescently-labeled primary antibodies. Phalloidin–Alexa Fluor 488 (1:40, Molecular Probes) was used to label filamentous actin (F-actin). Upon washing with PBS buffer, grown cells on glass coverslips were fixed with 4% paraformaldehyde in PBS buffer for 10 min at room temperature. Cells were subsequently washed with ice-cold PBS buffer and permeabilized for 5 min with 0.2% Triton X-100. Cells were washed thrice with PBS buffer and incubated with PBS containing 5% goat serum for 1 h at room temperature to block nonspecific binding sites of the antibodies. Primary antibodies were then added to the wells ad incubated overnight at 4 °C in the dark. Upon washing with PBS buffer, cells were incubated with phalloidin for 20 min. Samples were then covered using fluoroshield mounting medium with DAPI (ab104139, Abcam) and images were taken using an Olympus IX81 inverted fluorescence microscope (Olympus Corporation, Tokyo, Japan).

### Biocompatibility studies

#### MTT cytotoxicity assay

Cytotoxicity of XFS-targeting peptide solutions, as well as MP-peptide conjugates, was tested against hTM cells using MTT assay. Cells were plated in 24-well plates and upon reaching confluency they were treated with different concentrations of peptide solutions and particle-peptide conjugates. Cell proliferation was evaluated 24 h post-treatment using the Vybrant^®^ MTT cell proliferation assay kit (Molecular Probes).

#### Live/dead cell viability assay

Cell viability was further assayed using LIVE/DEAD^®^ viability/cytotoxicity kit (Molecular Probes). Monolayers of hTM cells were treated in triplicates with peptides alone in solution (1 mM) and in conjugation with MPs (100 µg). Cell viability was then assessed according to the manufacturer's protocol. The kit is based on calcein AM/ethidium homodimer-1 (EthD-1) system, where calcein AM detects cellular esterase activity of live cells and EthD-1 stains nuclei of dead cells. A similar live/dead assay was used to investigate the effect of MP-peptide conjugates on the viability of hTM cells in the presence of the rotating magnetic field. Cells grown in 24-well plate and treated with MP-peptide conjugates were placed under a rotating magnetic field for 1 h. The viability of hTM cells was then assessed as described above. A similar live/dead assay was carried out to evaluate the effect of the rotating magnetic field on cultured hTM cells. The plated cells in a 24-well cell culture plate were incubated with 100 µg MP-peptide conjugates. The plate was placed under a rotating magnetic field which was constructed using two attached cuboid-shaped permanent magnet (having a pole field strength of ~ 5000 G) to provide a larger field over the plate. The cells were treated with the rotating magnetic field for 10 min and then analyzed using live/dead assay as described before. For both MTT and live/dead assays one-way ANOVA test was used to analyse the significance of difference (p < 0.05) between the results.

### DNA fragmentation analysis

Effect of peptide-particle conjugates and peptide solutions on the induction of apoptotic DNA fragmentation was analyzed in hTM cells. Grown hTM cells in 24-well plates were incubated with 100 µg of peptide-MP complexes or 0.5 mM peptide solutions for 24 h at 37 °C. Cells were recovered from wells using a cell scraper. DNA was isolated and purified using DNeasy blood and tissue kit (Qiagen). Extracted DNA molecules from control and treated hTM cells were subjected to 2% agarose gel electrophoresis and visualized by SYBR green staining.

## Supplementary Information

**Additional file 1: Table S1. **Peptide enrichment during ex vivo biopanning against human lens capsules. **Figure S1. **Morphological analysis and immunofluorescence labeling of hTM Cells. (**A–C**) F-actin stained semi-confluent monolayer of cultured hTM cells. (**D–F**) hTM cells normally secreted fibronectin when grown on coverslips. (**G–L**) Grown cells expressed myocilin protein and laminin protein as a sign of normal phenotype. (**M**) Confluent monolayer of cultured cells showed contact inhibition having spindle-like shape, a typical characteristic of cultured hTM cells. (Sale bars = 50 µm).

## Data Availability

The data supporting the findings of this study are available from the corresponding author upon reasonable request.

## Abbreviations

XFS: Exfoliation syndrome
hTM cells: Human trabecular meshwork cells
hAH: Human aqueous humor
IOP: Intraocular pressure
MP: Magnetic particle
