# Supplementary material for: Towards preventing exfoliation glaucoma by targeting and removing fibrillar aggregates associated with exfoliation syndrome
Source: J Nanobiotechnology. 2022 Oct 27;20:459. doi: 10.1186/s12951-022-01665-6 (PMC9615239; doi:10.1186/s12951-022-01665-6)
Supplement: Supplementary file 1 — Additional file 1: Table S1. Peptide enrichment during ex vivo biopanning against human lens capsules. Figure S1. Morphological analysis and immunofluorescence labeling of hTM Cells. (A–C) F-actin stained semi-confluent monolayer of cultured hTM cells. (D–F) hTM cells normally secreted fibronectin when grown on coverslips. (G–L) Grown cells expressed myocilin protein and laminin protein as a sign of normal phenotype. (M) Confluent monolayer of cultured cells showed contact inhibition having spindle-like shape, a typical characteristic of cultured hTM cells. (Sale bars = 50 µm). [file 12951_2022_1665_MOESM1_ESM.docx]

**Table S1. Peptide enrichment during ex vivo biopanning against human lens capsules.**

| **Biopanning** | **Peptide** | **copies** | **% of sequences** | **Lens capsule type** |
| --- | --- | --- | --- | --- |
| Negative panning (subtraction) | --- | --- | --- | without XFS materials |
| Round 1 | --- | --- | --- | with XFS materials |
| Round 2 | p-LPS | 3/12 | 25 | with XFS materials |
|  | p-IPL | 4/12 | 33 |  |
| Round 3 | p-LPS | 11/52 | 21 | with XFS materials |
|  | p-IPL | 37/52 | 71 |  |

**Doubling time and immunohistochemistry of hTM cells**

The population doubling time for obtained hTM cells was determined to be 11.3 ± 2.1 hours, which is typical for fetal hTM cells [1]. Immunohistochemical analyses of hTM cells included evaluation of expression of fibronectin, myocilin, laminin, and actin. Is has been shown that actin microfilaments are mainly aligned parallel to the longitudinal axis of cultured hTM cells [2], which was also observed for our cells when stained with phalloidin dye (Fig. S1B). Expression of fibronectin protein was also confirmed in the cultured hTM cells (Fig. S1E). This proteins is one of the major extracellular matrix glycoproteins of hTM cells which is secreted as loosely aligned filaments on the cultured hTM cells surface or large bundles at the periphery of individual cells [3] Myocilin protein (or trabecular meshwork–inducible glucocorticoid response protein) is an highly expressed glycoprotein in the trabecular meshwork, and has been found within the cytoplasm of hTM cells when in association with extracellular matrix components [4][5]. The expression of myocilin is a key phenotypic characteristic of hTM cells and was observed in our cell culture (Fig. S1H). Expression of laminin is a marker to characterize cultured hTM cells [6], and was observed in immunohistochemical studies (Fig. S1K). The common morphology of hTM cells is the spindle-like shape which was also observed in monolayers of cultured hTM cells (Fig. S1M).

| 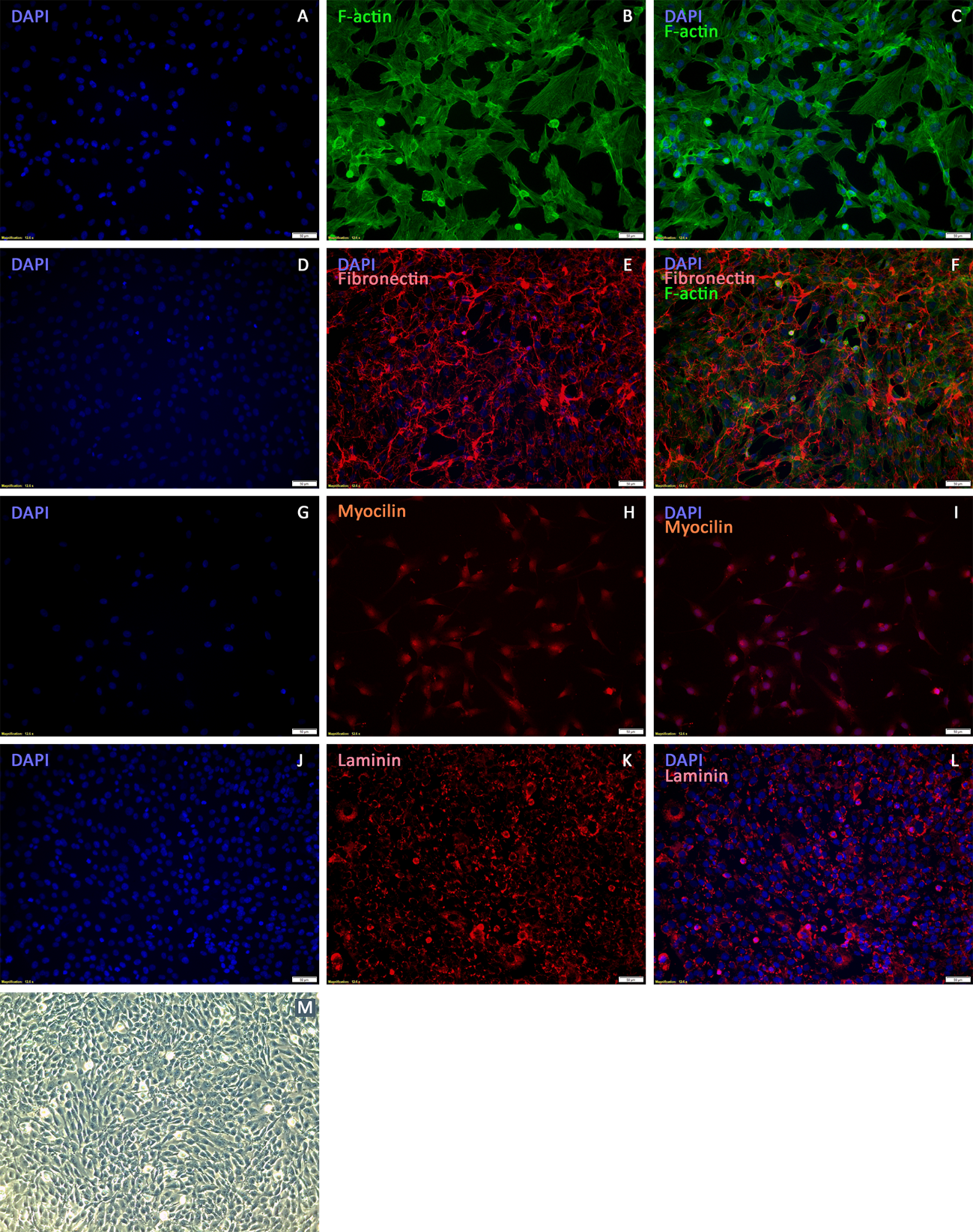 |
| --- |

**Figure S1. Morphological analysis and immunofluorescence labeling of hTM Cells.** (**A-C**) F-actin stained semi-confluent monolayer of cultured hTM cells. (**D-F**) hTM cells normally secreted fibronectin when grown on coverslips. (**G-L**) Grown cells expressed myocilin protein and laminin protein as a sign of normal phenotype. (**M**) Confluent monolayer of cultured cells showed contact inhibition having spindle-like shape, a typical characteristic of cultured hTM cells. (Sale bars = 50 µm).

**References**

[1] S. Lin, O.-T. Lee, P. Minasi, and J. Wong, “Isolation, culture, and characterization of human fetal trabecular meshwork cells.,” *Curr. Eye Res.*, vol. 32, no. 1, pp. 43–50, Jan. 2007.

[2] A. F. Clark *et al.*, “Dexamethasone alters F-actin architecture and promotes cross-linked actin network formation in human trabecular meshwork tissue.,” *Cell Motil. Cytoskeleton*, vol. 60, no. 2, pp. 83–95, Feb. 2005.

[3] H. T. Steely, S. L. Browder, M. B. Julian, S. T. Miggans, K. L. Wilson, and A. F. Clark, “The effects of dexamethasone on fibronectin expression in cultured human trabecular meshwork cells.,” *Invest. Ophthalmol. Vis. Sci.*, vol. 33, no. 7, pp. 2242–2250, Jun. 1992.

[4] J. R. Polansky *et al.*, “Cellular pharmacology and molecular biology of the trabecular meshwork inducible glucocorticoid response gene product.,” *Ophthalmol. J. Int. d’ophtalmologie. Int. J. Ophthalmol. Zeitschrift fur Augenheilkd.*, vol. 211, no. 3, pp. 126–139, 1997.

[5] E. R. Tamm, “Myocilin and glaucoma: facts and ideas.,” *Prog. Retin. Eye Res.*, vol. 21, no. 4, pp. 395–428, Jul. 2002.

[6] M. R. Hernandez, B. I. Weinstein, J. Schwartz, R. Ritch, G. G. Gordon, and A. L. Southren, “Human trabecular meshwork cells in culture: morphology and extracellular matrix components.,” *Invest. Ophthalmol. Vis. Sci.*, vol. 28, no. 10, pp. 1655–1660, Oct. 1987.
